# Changing Pattern of Dengue Virus Serotypes in Thailand between 2004 and 2010

**DOI:** 10.3329/jhpn.v30i3.12300

**Published:** 2012-09

**Authors:** Piyathida Pongsiri, Apiradee Themboonlers, Yong Poovorawan

**Affiliations:** Center of Excellence in Clinical Virology, Department of Pediatrics, Faculty of Medicine, Chulalongkorn University, Bangkok, Thailand

**Keywords:** Co-infection, Dengue virus, Prevalence, Serotype, Thailand

## Abstract

Dengue virus infection is a major concern in several countries, and more than 50 million people are infected worldwide each year. Thailand is one of the countries where people are susceptible to infection due to favourable geographical and environmental conditions. In this retrospective study, we reported the changing pattern of dengue virus serotypes during the period between 2004 and 2010. The following percentage prevalence showed different serotypes of dengue virus (DENV) predominant in respective years: DENV1 in 2004 (56.41%), DENV4 in 2007 (50%), DENV1 in 2008 (57.41%), and DENV3 in 2010 (38.7%). Moreover, the major serotypes were not stable as they showed a shift from one serotype to another. We also found co-infection with two different serotypes and reported the clinical manifestations, which were not different from infection with a single serotype. Co-infection with various serotypes may not necessarily cause more severe disease.

## INTRODUCTION

Dengue virus (DENV) is the most important arbovirus requiring high treatment cost. The virus also contributes to high mortality rate, particularly in tropical and subtropical countries. It has been a public-health concern for several years with an estimated 50 million infections worldwide per year, of which 250,000–500,000 cases turn to dengue haemorrhagic fever (DHF), leading to 25,000 deaths (1). Besides, the prevalence and severity of dengue virus infection has increased in its geographic distribution over the past few decades.

Dengue virus is a member of the family Flaviviridae and genus *Flavivirus*. It is an enveloped particle containing a 10.7 kb single-stranded RNA genome of positive polarity (2). Dengue virus has been divided into four serotypes: DENV1, DENV2, DENV3, and DENV4. It has been reported that certain serotypes of dengue virus can replace other serotypes. Besides, the four DENV serotypes can co-circulate and co-infect individual humans, which appears to be a common feature in some outbreaks (3).

Dengue virus causes varying clinical symptoms ranging from dengue fever and dengue haemorrhagic fever (DHF) to dengue shock syndrome (DSS) according to the World Health Organization criteria (4). For example, people infected by dengue virus may be asymptomatic or the infection could be life-threatening. Patients with dengue fever show onset of fever, headache, rash, and arthralgia. DHF patients present with more severe symptoms, such as higher fever, haemorrhagic manifestations (leukopenia, thrombocytopenia, atypical lymphocytosis), and liver involvement. Clinical signs of plasma leakage, such as pleural effusion, ascites, or hypoproteinaemia constitute another criterion to differentiate DHF from dengue fever. DHF patients have to be monitored closely because it may lead to a life-threatening condition, such as disseminated intravascular coagulation and dengue shock syndrome.

Morbidity from dengue virus infection can be attributed to any serotype, with some reports indicating that multiple infections with different serotypes may contribute to increased disease severity (5). DENV1-3 seemed to be responsible for several outbreaks in Brazil and the USA (6). Each serotype of dengue virus may have a different genetic component associated with virulence but there has been no solid confirmation. Another possibility is that antibody-dependent enhancement (ADE) may be associated with severity in that primary infection induces insufficient protection and assists entry of virus into cells, resulting in higher peak viral titres (7).

As Thailand is one of the countries that reported dengue virus infections, enhancing our knowledge on viral dissemination patterns would be beneficial. In this study, we have investigated infection with various serotypes in Thai patients between 2004 and 2010. Moreover, we report the co-infection of dengue virus with information on clinical symptoms. We hope that our report on the changing pattern of dengue virus serotypes, along with co-infection, may provide important information on epidemiology, prevalence, and virulence of dengue infection.

## MATERIALS AND METHODS

The protocol of the study was reviewed and approved by ethics committee of the Institutional Review Board (IRB), Faculty of Medicine, Chulalongkorn University, Thailand. We also included data from two previous reports (8,9). All the study subjects remained anonymous, and permission was granted by the director of the hospital.

### Serum samples

Serum samples were collected from patients within 1-7 day(s) after the onset of fever. The samples were collected from patients who had fever, headache, rash, or myalgia/arthralgia-positive tour­niquet test result, thrombocytopenia or plasma leakage since they were likely to be infected by dengue virus according to WHO criteria (4). We have investigated 100 samples from 2004, 104 samples from 2007, 118 samples from 2008, and 113 samples from 2010 by RT-PCR. The samples showing positive results in RT-PCR from the year 2004 (n=39), 2007 (n=32), 2008 (n=84), and 2010 (n=30) were subsequently subjected to sequencing to obtain information on the nucleic acid details and were analyzed by BLAST to determine the type. Unfortunately, we had no specimens for dengue serotype examination from the year 2005, 2006, and 2009.

### RNA extraction and reverse transcription

RNA was extracted from serum samples, using the Viral Nucleic Acid extraction kit (RBC bioscience, Taipei, Taiwan) according to the manufacturer's protocol. The extracted RNA was reverse-transcribed into cDNA by ImProm-II^TM^ Reverse Transcriptase (Promega, Medison, WI).

### Semi-nested RT-PCR

The first and second amplification steps were performed in an Eppendorfthermalcycler (Eppendorf, Hamburg, Germany). After initial denaturation at 95 ^o^C for 2 minutes, the amplification reaction comprised 40 cycles of denaturation at 95 ^o^C for 25 seconds, annealing at 50 ^o^C for 35 seconds and extension at 72 ^o^C for 1 minute and was concluded by a final extension step at 72 ^o^C for 5 minutes and a final hold step at 25 ^o^C for 5 minutes. The primer targeting the 3’ non-translated region has been previously described (8). The estimated amplicon-sizes of different serotypes of dengue virus were 434 bp, 420 bp, 417 bp, and 358 bp for DENV1, DENV2, DENV3, and DENV4 respectively.

### Post-amplification step and sequence analysis

The PCR products were subjected to electrophoresis on a 2% agarose gel stained with ethidium bromide and visualized under UV light. The PCR products of the expected sizes were excised from the gel and purified using the gel purification kit (RBC bioscience, Taipei, Taiwan). The purified DNA was subjected to direct sequencing (1st base, Salangor, Malaysia), and the obtained nucleotide sequences were analyzed using the BLAST program available at the GenBank database. Serotypes were compared based on the relationship between genomic RNA of dengue virus and the serotype (10).

### Statistical analysis

The results were compared statistically by using chi-square (StatCalc version 6). The p value of <0.05 indicated statistically significant differences. Each serotype was determined independently. We calculated the p value of each pair in different years, such as between 2004 and 2007, 2004 and 2008, and 2004 and 2010. Yearly data for 2004, 2007, 2008, and 2010 were also compared.

## RESULTS

### Comparative serotype analysis of dengue virus

In 2004, a total of 39 positive samples were analyzed showing that the 2004 outbreak included DENV1 (56.4%, n=22), DENV2 (28.2%, n=11), DENV3 (5.1%, n=2), and DENV4 (10.3%, n=4). Two years later, in 2007, the results included DENV1 (3.1%, n=1), DENV2 (25%, n=8), DENV3 (21.9%, n=7), and DENV4 (50%, n=16). When compared DENV1 between 2004 and 2007, the percentage of DENV1 in 2007 was significantly lower compared to the 2004 outbreak (p<0.05). However, when compared DENV1 between 2007 and 2008, the result showed an exponential increase to 57.1% (n=48) (p<0.05) and then a decrease to 25.8% (n=8) in 2010.

When compared between 2004 and 2007, the DENV4 percentage increased significantly from 10.26% (n=4) to 50% (n=16) (p<0.05). After that, in 2008 and 2010, DENV4 percentage declined to 11.9% (n=10) and 6.45% (n=2) respectively.

In 2004, DENV2 was 28.21% (n=11), which is quite similar to the proportion of DENV2 in 2007 (25%, n=8). DENV2 still showed lower proportion in 2008 (9.52%, n=8). In 2010, however, the percentage of DENV2 considerably increased up to 38.7% (n=12) (p<0.05), which also seemed to be predominant during this year, although there was no obviously-predominant serotype amounting to 50% in 2010.

**Fig. 1.**
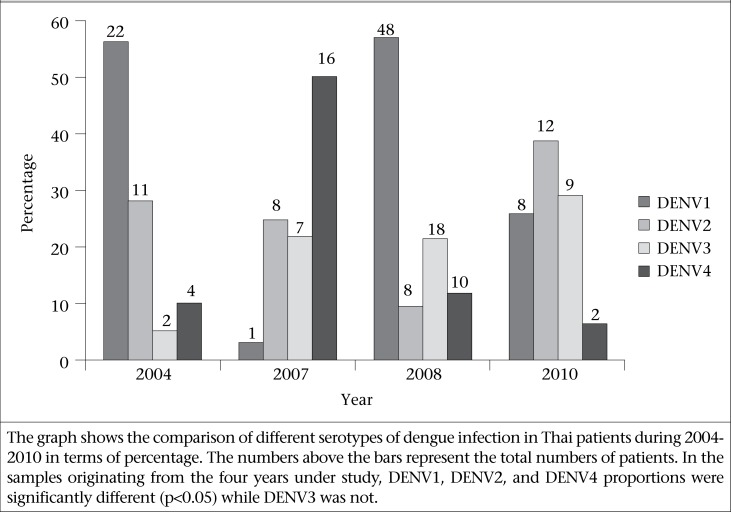
Comparison of different serotypes of dengue-infection in Thai patients during 2004-2010

The DENV3 proportion in 2004 was 5.13% (n=2) and then increased to 21.9 % (n=7) in 2007 and showed insignificant changes in 2008 and 2010 with 21.4% (n=18) and 29.03% (n=9) respectively. The details are shown in Fig. 1. In this study, different serotypes of dengue virus throughout the study years have not shown significant differences in severity of illness due to the predominant serotypes. The common illnesses were fever and rash regardless of why serotype of dengue virus infected the patients. We found one patient co-infected with DENV3 and DENV4 in 2010.

### Clinical presentation of patient co-infected with two dengue serotypes

**Fig. 2.**
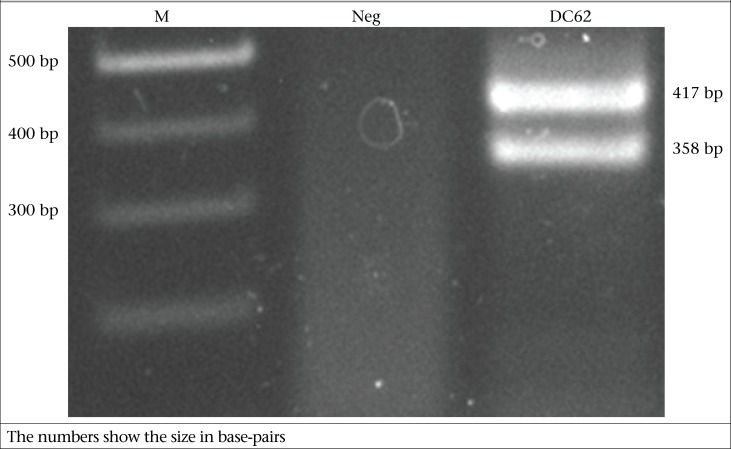
Gel-electrophoresis of PCR products after semi-nested PCR showing DENV3 of 417 bp and DENV4 of 358 bp

In September 2010, a 3-year old female patient from a rural area in the northeast of Thailand came to the hospital and was admitted on day 5 after onset of febrile illness. Her symptoms were epitaxis, malaise, loss of appetite, nausea, and vomiting. On the day of admission, she had a high-grade fever without any signs of respiratory tract infection. Routine laboratory test showed haematocrit 33.3%, WBC 6700 mm^3^, N 23%, L 52.9%, M 17.4%, B 6.1%, atypical lymphocyte 3.9%, and decreased platelets on blood smear. She recovered and was discharged after 3 days of hospitalization. Dengue fever was diagnosed based on detectable IgG and IgM specific to dengue virus through ELISA. The diagnosis was confirmed by detection of DENV RNA with semi-nested multiplex RT-PCR. The results showed evidence of co-infection with two serotypes of dengue virus—DENV3 and DENV4 (Fig. 2). The PCR results were confirmed by direct sequencing (accession number JF737997 for DENV3 and JF737996 for DENV4).

## DISCUSSION

Our retrospective study has shown that the serotypes of dengue virus spreading in Thailand were fluctuating, which is indicative of the dynamics of virus population. Since 2000-2001, DENV1 seemed to be the main serotype spreading in the Pacific region, including Thailand (11). Beginning in 2004, DENV1 showed its obvious predominance. In 2007, the main serotype was DENV4 and interestingly, in the following year (2008), the rapid displacement of DENV1 was observed. This report shows that serotype predominance can shift from one to another. This study is in agreement with another study demonstrating that DENV1 was replaced by DENV4 (12). Some DENV serotypes, for example DENV1, may cause more severe illness. As the majority of the samples in this study were obtained from hospitalized patients, the results may not actually reflect the predominant serotype in a given year.

The antibodies specific to each serotype might be responsible for the dissemination pattern. Although primary infection provides cross-protection among dengue virus, it is not sufficient to completely protect patients from secondary infection, especially when the patient gets the second infection with a serotype different from that of the primary infection. There is also antibody-dependent enhancement (ADE) that mediates the entry of dengue virus into the host cell and can lead to a severe condition, particularly cross-reacting antibodies. Besides, the major sites of DENV replication are monocytes and macrophages, and when the immune system cross-reacts, it might mediate entry of virus into the Fc-bearing cell, and eventually increase replication of virus (13). Dengue infection also affects T-cell function and triggers an increase in apoptosis which leads to a more severe condition (14). Moreover, there was asymmetric competition among dengue serotypes. Upon testing DENV2 and DENV4, the results showed that the competition among DENV strains in cultured mosquito cells can cause a significant decrease in sizes of peak virus population. The respective percentage of each DENV serotype may be due to the competition among serotypes as well (3).

Co-circulation and co-infection with various dengue serotypes are common as has been reported from India and Brazil (15). Our study has also shown co-circulation in the course of outbreaks. The difference in the ability of different serotypes of dengue virus to infect the host may also be due to genomic differences. Patients with multiple-serotype infection may present with a more severe condition than those with single-serotype infection and has been reported to progress towards DHF (16). However, our report has shown that such a hospitalized patient may also display more common conditions and can recover within 6 days from the onset of fever.

### Conclusions

A single serotype of dengue virus may not be able to circulate in any given endemic area. The predominant DENV serotype has been continuously shifted from year to year depending on the herd immunity to DENV and competition among serotypes. Co-infection with various serotypes of dengue virus may not necessarily lead to a more severe condition or DHF/DSS.

## ACKNOWLEDGEMENTS

This study was supported by the Center of Excellence in Clinical Virology, Chulalongkorn University, CU Centenary Academic Development Project, King Chulalongkorn Memorial Hospital, the National Research University Project of CHE (HR1155A) and the Ratchadaphiseksonphot Endowment Fund. We would like to thank the staff and nurses of Chumphae Hospital, Khon Kaen province, Thailand, for collecting the clinical data and specimens. Besides, we would like to express our gratitude to Ms Petra Hirsch for reviewing the manuscript.
